# Developing next generation immunomodulatory drugs and their combinations in multiple myeloma

**DOI:** 10.18632/oncotarget.27973

**Published:** 2021-07-20

**Authors:** Anjan Thakurta, William E. Pierceall, Michael D. Amatangelo, Erin Flynt, Amit Agarwal

**Affiliations:** ^1^Translational Medicine, Bristol Myers Squibb, Summit, NJ, USA; ^2^Global Medical Affairs, Bristol Myers Squibb, Summit, NJ, USA

**Keywords:** multiple myeloma, combinations, immunomodulatory agents, apoptosis, protein degradation

## Abstract

Multiple Myeloma (MM) is an incurable malignancy with current treatment choices primarily comprising combination regimens implemented with a risk-adapted approach. Cereblon (CRBN)-targeting immunomodulatory agents (IMiDs^®^) lenalidomide (LEN) and pomalidomide (POM) play a central role in combination regimens due to their pleiotropic antitumor/immunomodulatory mechanisms that synergize with many anti-myeloma approved or developmental agents. Currently, more potent next generation cereblon E3 ligase modulators (CELMoDs^®^) - iberdomide (IBER) and CC-92480 are in clinical development. With an expanding number of active agents/therapeutic modalities and a myriad of combinatorial possibilities, physicians and drug developers share an opportunity and challenge to combine and sequence therapies to maximize long-term patient benefit. Understanding drug mechanisms and their application in combination settings as well as the unique disease biology considerations from newly diagnosed (NDMM), relapsed/refractory (RRMM), and maintenance settings will be vital to guide the development of future MM therapies centered on a backbone of IMiD or CELMoD agents. Key aspects of drug activity are critical to consider while evaluating potential combinations: direct antitumor effects, indirect antitumor cytotoxicity, immune surveillance, and adverse side effects. In addition, the treatment journey from NDMM to early and late MM relapses are connected to genomic and immune changes associated with disease progression and acquisition of resistance mechanisms. Based on the types of combinations used and the goals of therapy, insights into mechanisms of drug activity and resistance may inform treatment decisions for patients with MM. Here we focus on the evolving understanding of the molecular mechanisms of CRBN-binding drugs and how they can be differentiated and suggest a strategic framework to optimize efficacy and safety of combinations using these agents.

## LEARNINGS FROM THE BENCH

**Figure 1:**
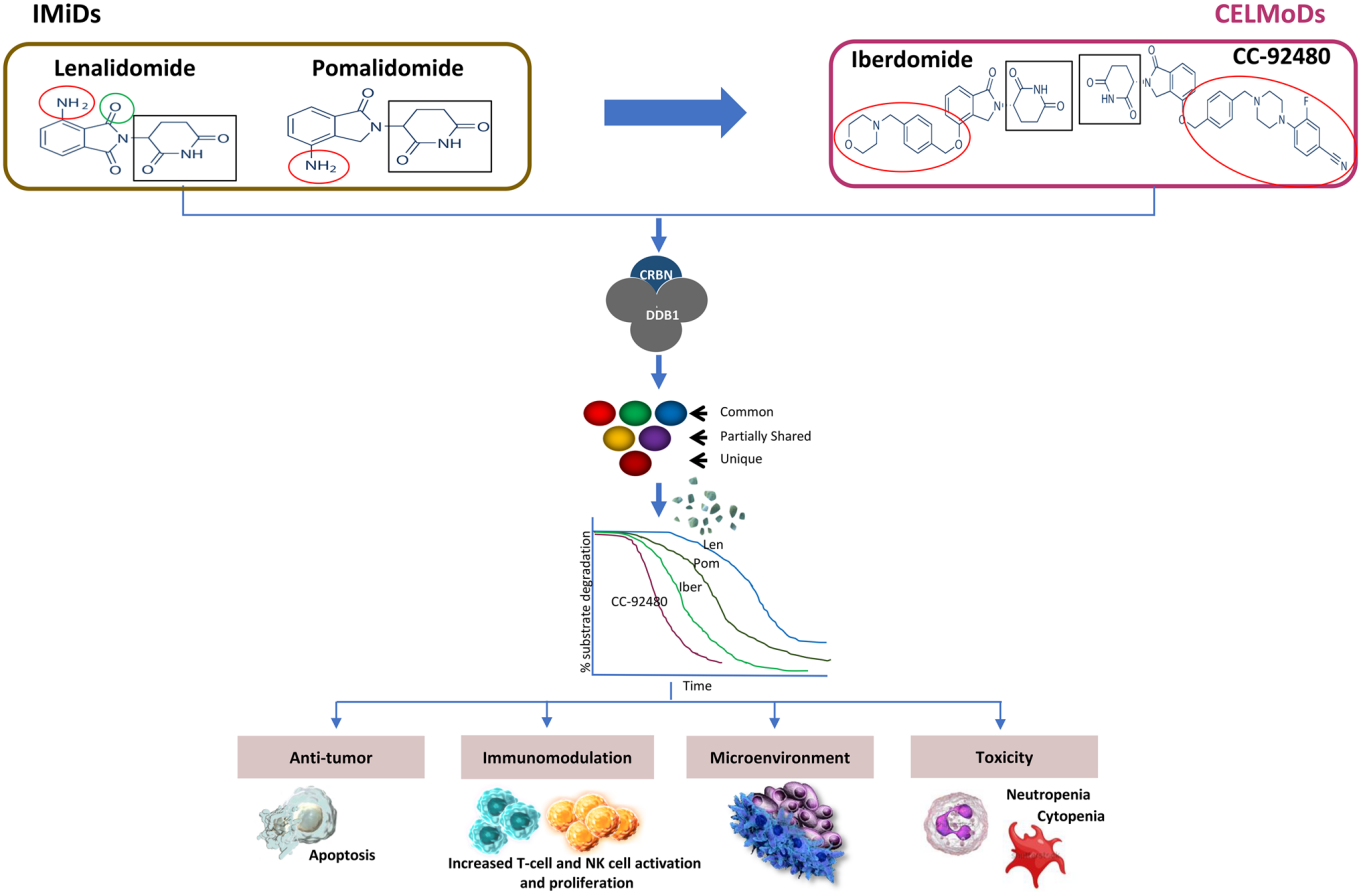
Differentiation of IMiDs and CELMoDs. Shown are structures of IMiDs and CELMoDs in myeloma clinical development. Mechanism of action differentiation is primarily derived from the physical interactions with the CRBN/DDB1 complex that may exert regulation of unique and overlapping substrate preference and degradation kinetics and modulation of distinct cell types.

IMiD/CELMoD compounds bind CRBN, a substrate receptor of the Cul4A/DDB1/Roc1 (Cul4A^CRBN^) E3 ligase complex, and trigger recruitment, polyubiquitination and subsequent degradation of substrate proteins. A simple framework (Figure 1) underscores critical parameters that capture the diversity of the downstream biochemical or biological effects of these drugs in target cell types, via key common molecular steps. Chemically, IMiD and CELMoD agents share glutarimide rings for binding to the tri-tryptophan pocket of cereblon, and isoindolinone rings that interact with cereblon and substrates (e.g. ikaros, aiolos, etc.). However, CELMoD structures are extended relative to those of IMiDs, containing additional phenyl and morpholino moieties enabling enhanced interactions with cereblon or substrates [1, 2]. While both LEN and POM bind CRBN with similar affinity (K_d_~1.0–1.5 uM) [3, 4], POM is more potent and efficient in substrate degradation and retains antitumor activity against LEN-resistant cell lines. Notably, IBER and CC-92480 bind CRBN with ~10-20-fold higher affinity and induce more potent and efficient degradation of Ikaros and Aiolos as compared to LEN/POM [4, 5]. The superior CRBN-binding affinity of CELMoD agents (CC-92480>IBER) compared to IMiDs is one of the key features that differentiates these compounds. This potency difference may explain the superior cell autonomous activity of CELMoD compounds and their ability to function with lower levels of CRBN and/or in IMiD-resistant backgrounds due to CRBN dysfunction (e.g., mutations, splice variants etc.). Notably, IBER was shown to retain activity in POM-resistant cell lines with reduced CRBN expression [6] and CC-92480 was active against CRBN mutants expressed in cell lines which are normally resistant to POM [5].

IMiD and CELMoD agents have shared as well as unique substrates; their downstream cellular effects may be differentiated by the presence, abundance of, and relative preference for these key substrate proteins of functional consequence (Figure 1). Among the various substrate proteins described, transcription factors Ikaros and Aiolos are directly linked to the activity of these agents. Ikaros and Aiolos regulate lymphoid cell development and homeostasis and are required for the growth and survival of B- and plasma cells. [7–10]. Importantly, the rate of degradation of Ikaros and Aiolos and subsequent downregulation of IRF4 and c-MYC expression correlates with downstream pharmacological effects of IMiD and CELMoD compounds in MM cell lines [7, 11–13]. Faster degradation of substrates by IBER and CC-92480 is associated with more potent anti-myeloma activity and immune stimulatory activity as compared to LEN/POM (Figure 1) [5, 14]. Similar to their activity in MM cells, IMiD/CELMoD modulation of substrate degradation occurs in immune subpopulations, manifesting in diverse biological effects. Specifically, these compounds act on lymphoid (e.g., T-, B-, NK-, Treg cells), myeloid (e.g., monocyte/macrophage, myeloblasts), and stromal cells, leading to immunomodulatory effects by expanding, activating, or inhibiting the functions of these cells. For instance, IMiD-driven substrate degradation in T cells leads to immune activation via IL-2 up-regulation [15, 16]. *In vitro* studies with LEN and POM demonstrated broad co-stimulatory effects in primary human T and NK cells, including induction of proliferation, cytokine secretion and cytotoxicity [17–19]. Added to the direct antitumor activities of these agents, the immunomodulatory effects could result in synergistic activities with other anti-myeloma therapies.

Preclinical models showed enhanced immune co-stimulatory activities of IBER when compared to LEN and POM, including increased IL-2 secretion and granzyme-B degranulation in stimulated peripheral blood mononuclear cells (PBMCs) and enhanced immune-mediated killing of MM cells [6, 20]. Furthermore, in combination studies, IBER treatment increased the activity of daratumumab (DARA) more than IMiD compounds, and also enhanced the anti-tumor activity of CAR-T based therapies [21–23]. The improved CRBN-modulating activities are also manifest by CC-92480, exhibiting increased potency against immune cells and enhanced immune-based activity of therapies like DARA [5, 24]. While the potent degradation in Ikaros and Aiolos protein induced by IBER and CC-92480 represents the dominant mechanism for their pharmacological effects, other substrates may play a role in differentiating these agents. Taken together, the enhanced potency and improved immunostimulatory properties of CELMoD therapies may result in clinical activity in NDMM or RRMM patient molecular segments including those resistant/refractory to IMiD agents [6, 25].

## LEARNINGS FROM THE BEDSIDE

Biomarker analyses of tumor and immune samples from clinical trials in RRMM settings demonstrate mechanistic differences among IMiD and CELMoD therapies. Loss of CRBN expression was initially suggested as a mechanism of resistance to LEN [10, 13]. Mutations in CRBN are rare in NDMM patients [26]. However, analysis of clinical samples from RRMM patients has shown an increase in CRBN aberrations as patients relapse on IMiD-based treatments [27, 28]. Acquired resistance due to CRBN aberrations can be grouped into four main buckets: decrease in gene expression, copy-loss and other structural variants involving the CRBN gene locus Ch 3p26, changes in mRNA splicing and gene mutations [25]. In a large study of RRMM patients treated with IMiD therapies, 20% of LEN-refractory patients and 30% of POM-refractory patients had a CRBN-related abnormality [25]. Cumulatively, acquisition of these aberrations was correlated with poor clinical outcome with a subsequent IMiD-containing regimen. While analyses of patient samples treated with the CELMoD agents are ongoing, a mechanistic hypothesis to be tested is whether next generation CELMoD therapies overcome clinical resistance caused by CRBN aberrations. CELMoD therapies exert more efficient substrate degradation in patients with low CRBN levels or a high proportion of CRBN splice variants lacking the IMiD-binding domain (e.g. CRBN exon 10 deletion) relative to main CRBN transcript [29]. Similarly, CELMoD agents with higher binding affinity and larger size may increase physical interaction with CRBN surface to overcome destabilizing effects of mutations on CRBN protein structure and function. Initial analysis of the CC-220-MM-001 study (NCT02773030), where over 80% of patients are IMiD-refractory, has shown a significant enrichment in patients with CRBN abnormalities. Interestingly, Aiolos and Ikaros protein degradation is maintained in these IBER-treated patients. Trial data provide support that CELMoD agents are active in MM patients refractory to IMiD agents with dysregulated CRBN [30].

Immunomodulatory data from patients treated with IMiD therapies showed an increase in bone marrow T lymphocytes [31, 32]. More recently, modulation of innate and adaptive immunity by POM-based regimens has become more defined and correlated with clinical antitumor effects [33, 34]. Notably, POM plus dexamethasone (POMdex), POMdex with cyclophosphamide [35], or POMdex with DARA [33] consistently showed decrease in naïve T cells with concurrent increase in effector memory and activated/proliferating T cells. Notably, this enrichment of a differentiated and cytotoxic T cell phenotype by POM-based regimen still occurs in patients refractory to LEN [33]. Immunophenotyping in RRMM patients treated with IBER or CC-92480 [36, 37] alone and in combinations has shown similar enhancements even in the later line patients treated thus far, including reduction in mature B-cells and naïve T cells and increase in proliferating NK cells and effector memory T cells. The immune changes were dose-dependent and most pronounced when exposed to higher doses. This insight may be utilized to provide rationale for dose and schedule optimization to deliver specific immune effects in combination with other immunotherapies, even among distinct therapeutic modalities such as bispecific antibodies and CAR-T cells.

## MECHANISM-DRIVEN ADVERSE EVENTS

IMiD therapies have both overlapping and distinct toxicities. Neutropenia and thrombocytopenia are common serious adverse events (SAE) in patients treated with IMiD compounds. Previous studies with LEN plus dexamethasone (LENdex) showed 30–40% grade 3/4 neutropenia in RRMM patients [38, 39]. In POMdex treated patients, the incidence of grade 3/4 neutropenia was ~50% [40, 41]. Recent studies on molecular mechanisms of IMiD agent-mediated myelosuppression showed LEN and POM reduced PU.1 expression in CD34^+^ cells, leading to maturation arrest of myeloid precursors, and subsequent neutropenia [42, 43]. The PU.1 decreased expression in CD34^+^ cells following IMiD exposure was found to be Ikaros-mediated and the subsequent maturation arrest at the promyelocyte stage shown to be reversible [44]. In additional myelosuppression studies, the reduction of GATA1 expression by IMiD agents led to arrest in megakaryocyte precursors impacting thrombocytopenia [42, 43].

To date, with limited clinical experience grade 3/4 neutropenia rates with IBER plus dexamethasone (IBERdex) are ~30% [30]. Similarly, Grade 3/4 thrombocytopenia in patients treated with IBERdex was reported to be ~12% [30]. With more clinical data the overall safety profile of IBER will be established that would provide better understanding of its effect on myelosuppression (Figure 1). This mechanistic understanding of SAEs may also help to inform understanding of overlapping toxicities in combination therapies, which can be dose-limiting for CD38-directed antibodies and proteasome inhibitors (PIs) with IMiD/CELMoD-based regimens.

## CLINICAL EVOLUTION FROM IMID TO CELMOD THERAPIES

The foundational role of IMiD agents in the treatment of MM evolved from several attractive features of these drugs making them ideal partners for combination therapy. Initial studies with LENdex showing activity in the RRMM setting allowed for the addition of several agents to this backbone: carfilzomib, DARA, elotuzumab (ASPIRE, POLLUX, ELOQUENT) [45–47]. Similarly, the demonstration that LENdex improved progression-free survival (PFS) compared to previously established standard therapies such as melphalan, thalidomide, and prednisone (MPT) in NDMM transplant ineligible patients paved the way to investigate combination studies with bortezomib and DARA (SWOG S0777, MAIA) leading to new standards of care in that patient setting [48, 49]. While these advancements have significantly improved outcomes of MM patients, there remains a need to improve progression free survival (or durability of response) in NDMM transplant ineligible patients. While these lenalidomide-based regimens improved PFS, there is a significant impact on quality of life of patients. POM was developed to be able to rescue responses in patients who relapsed on LEN. Several studies with POMdex demonstrated improved activity in RRMM patients previously treated with LEN [50, 51]. Evidence of the ability to sequence POM after LEN showed the possibility to overcome resistance to one generation of this class of drugs with more potent agents. Confirmation of improved activity of POMdex in RRMM patients allowed assessment of successful combinations of POMdex with bortezomib (OPTIMISMM) [52], elotuzumab (ELOQUENT-3) [53], isatuximab (ICARIA-MM) [54], and daratumumab (APOLLO) in phase 3 studies. Early studies with IBER and CC-92480 are showing similar trends. The phase I studies of IBER and CC-92480 have included 85–90% MM patients who are refractory to IMiDs (NCT02773030, NCT03374085). Responses observed in this group of patients demonstrates that CELMoD agents may display broader clinical activity among a broader patient group less likely to exhibit benefit to IMiD therapeutics. This proof of concept should allow for studies of CELMoD combinations with approved and novel agents compared to IMiD regimens.

## INFORMING RATIONAL COMBINATIONS: LOOKING FOR A SWEET SPOT

**Figure 2:**
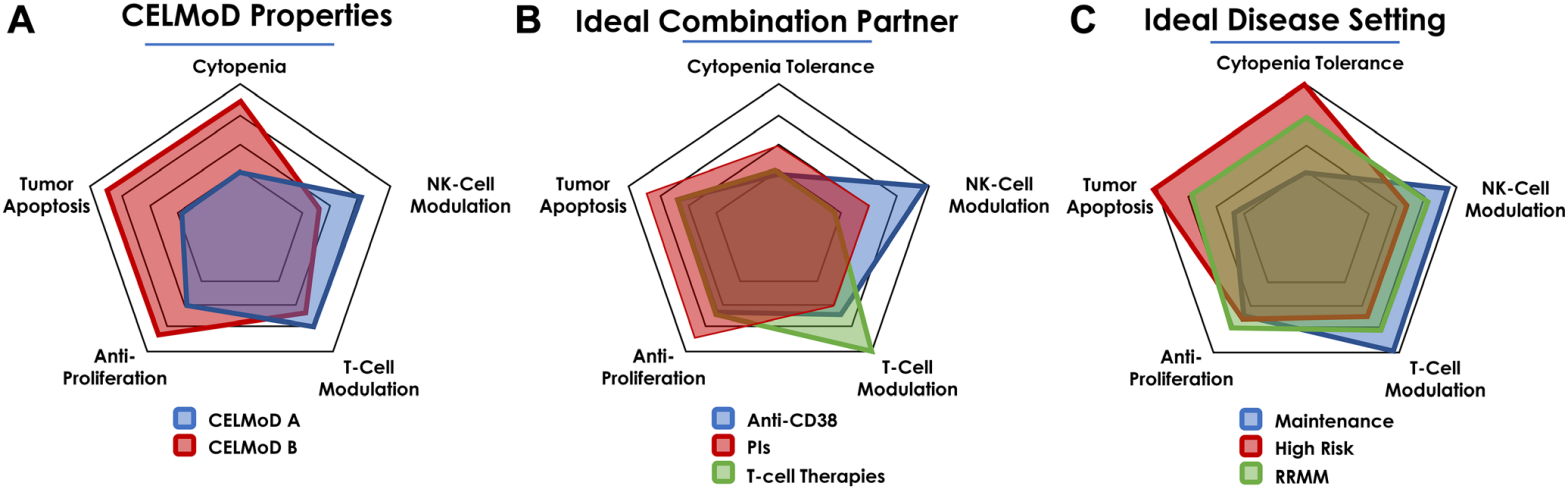
Hallmarks of CELMoDs desirable traits and rational combinations in MM settings. (**A**) CELMoDs may display distinct MoA by differential modulation of distinct cell types. (**B**) The ability to differentially act on distinct cell types may provide rational combinations based on enhanced potencies and mitigated safety signals. (**C**) The properties may be best utilized for ideal positioning and sequencing in specific lines of therapy of the patient journey.

How might we propose a rational combination approach for the CELMoD therapies in view of cell autonomous (e.g., antiproliferative, pro-apoptotic), immunomodulatory (e.g., NK, T cell stimulation) and adverse effects of these agents? Figure 2 depicts the relative differences in these properties of two hypothetical agents CELMoD A and B, their partners (e.g., CD38 antibody, PI), and different disease settings (NDMM, RRMM) for combination scenarios based on the overall effect based on these properties.

The design and selection of the new CELMoD agents relative to previous generation IMiDs has ensured superior pro-apoptotic and anti-proliferative activities while concurrently enhancing immunomodulation through augmentation of the NK cell compartment and T cell proliferation/activation. Additionally, with these more potent agents, attention has also been directed towards controlling dose-limiting cytopenias, which are known safety signals associated with IMiD agents (Figure 2A). CELMoD therapies may be differentiated on these aspects, and thus positioning these agents relative to overlapping mechanisms of disease control and safety should be considered in their clinical development.

Rational combination approaches for CELMoDs and approved agents should be based on the overlapping mechanisms of each contributing therapeutic modality. For instance, PIs are known as immunosuppressive agents that exert anticancer activity by proapoptotic effects. An ideal CELMoD therapy may add to the pro-apoptotic activity while counterbalancing the immunosuppressive nature of the PI (Figure 2B). In Figure 2B, Anti-CD38 not only promotes depletion of CD38 expressing plasma cells but also NK cells. CELMoD agents may counterbalance the NK cell pool with a preference for a CELMoD therapy in which cytopenias (such observed with POM-DARA-dex) may be less pronounced.

Lastly, the hallmark traits of the CELMoD agents may also provide guidance for settings and patient subpopulations in which clinical benefit may be derived. For instance, a CELMoD with enhanced antitumor activity but no appreciable increase in safety signal may be well positioned in NDMM patients or as maintenance treatment. Alternatively, patients in the RRMM setting that may display poor prognostic features may benefit from an agent with dialed-up apoptotic activity (Figure 2C). Clinical trials evaluating combination regimens with CELMoDs are either planned or currently underway. These studies would allow for correlative analysis to understand how these combinations build upon previous IMiD combinations.

## CONCLUSIONS

Interrogating the molecular mechanisms of action of IMiD/CELMoD agents over the past several years has brought to light common and unique properties of these agents in preclinical and clinical settings. Further understanding of two key areas about the properties of these agents is critical to drive their future development. First, better understanding of the molecular mechanistic features of CELMoD compounds will help differentiate them clinically from IMiD agents and from one another. The basic framework described here provides a roadmap for guidance. Analysis of biomarkers from clinical studies will complement this effort. Second, and more importantly, learning from clinical studies to form an integrated view of the desirable “sweet spot” profile (Figure 2) for combined efficacy and safety, should enable a more precise combination therapeutic approach. As our understanding of the mechanisms of resistance to these agents become more refined, in the future, we may be able to apply the right combination therapy for the right myeloma patients, and appropriately sequence them for maximum patient benefit.
